# Proteomic and Bioinformatic Investigation of Altered Pathways in Neuroglobin-Deficient Breast Cancer Cells

**DOI:** 10.3390/molecules26082397

**Published:** 2021-04-20

**Authors:** Michele Costanzo, Marco Fiocchetti, Paolo Ascenzi, Maria Marino, Marianna Caterino, Margherita Ruoppolo

**Affiliations:** 1 Department of Molecular Medicine and Medical Biotechnology, School of Medicine, University of Naples Federico II, 80131 Naples, Italy; michele.costanzo@unina.it; 2 CEINGE—Biotecnologie Avanzate S.C.Ar.L., 80145 Naples, Italy; 3 Department of Science, University Roma Tre, 00146 Rome, Italy; marco.fiocchetti@uniroma3.it (M.F.); paolo.ascenzi@uniroma3.it (P.A.); maria.marino@uniroma3.it (M.M.)

**Keywords:** neuroglobin, 17β-estradiol, label-free proteomics, bioinformatics, pathway analysis, oxidative stress, apoptosis, mitochondria, breast cancer cells

## Abstract

Neuroglobin (NGB) is a myoglobin-like monomeric globin that is involved in several processes, displaying a pivotal redox-dependent protective role in neuronal and extra-neuronal cells. NGB remarkably exerts its function upon upregulation by NGB inducers, such as 17β-estradiol (E2) and H_2_O_2_. However, the molecular bases of NGB’s functions remain undefined, mainly in non-neuronal cancer cells. Human MCF-7 breast cancer cells with a knocked-out (KO) *NGB* gene obtained using CRISPR/Cas9 technology were analyzed using shotgun label-free quantitative proteomics in comparison with control cells. The differential proteomics experiments were also performed after treatment with E2, H_2_O_2_, and E2 + H_2_O_2_. All the runs acquired using liquid chromatography–tandem mass spectrometry were elaborated within the same MaxQuant analysis, leading to the quantification of 1872 proteins in the global proteomic dataset. Then, a differentially regulated protein dataset was obtained for each specific treatment. After the proteomic study, multiple bioinformatics analyses were performed to highlight unbalanced pathways and processes. Here, we report the proteomic and bioinformatic investigations concerning the effects on cellular processes of NGB deficiency and cell treatments. Globally, the main processes that were affected were related to the response to stress, cytoskeleton dynamics, apoptosis, and mitochondria-driven pathways.

## 1. Introduction

Neuroglobin (NGB), discovered in 2000, is a vertebrate oxygen-binding protein belonging to the superfamily of globins [1]. Despite its name referring to the locus where it was originally discovered, namely, the neurons of the central and peripheral nervous system, NGB was detected in other tissues and organs as well [2]. Evidence showed that NGB accumulation significantly protects the brain from hypoxic/ischemic and oxidative stress injury, while NGB reduction exacerbates tissue damage. Human NGB overexpression has been suggested to shield neurons from mitochondrial dysfunctions and neurodegenerative disorders and to play a protective role in cancer cells [2]. The increasing interest in the functional roles played by NGB fits with the evidence that NGB is a stress-induced globin whose accumulation has been related to a positive modulation of cell viability during hypoxia and oxidative stress events [3,4]. The role of NGB accumulation as a protective shield of cancer cells to cope with their stressful environment has been demonstrated in 17β-estradiol (E2)-sensitive cancer cells [5]. In breast cancer cells, E2 induces NGB upregulation and accumulation into mitochondria via the estrogen receptor α (ERα)-activated protein kinase B (AKT) pathway; this leads to the inhibition of the proteasome- and lysosomal-mediated NGB degradation, augmenting the phosphorylation of the nuclear transcription factor CREBP that is responsible for the transcription of the *NGB* gene [6]. Mitochondrial accumulation of NGB in breast cancer cells counteracts the trigger of apoptosis induced by oxidative stress [7]. Intriguingly, NGB silencing renders MCF-7 cancer cells more prone to the apoptosis induced by the chemotherapeutic agent Paclitaxel, even in the presence of E2 [8]. Of note, in H_2_O_2_-treated breast cancer cells, NGB accumulation is detected mainly in the cytosol and released in the tumor microenvironment, where it acts as an autocrine/paracrine factor that can communicate cell resilience against oxidative stress and chemotherapeutic treatment [9]. These data indicate that NGB accumulation could drive breast cancer cells to different destinies depending on the NGB inducers, thus enlarging the functional role of this globin. Nowadays, the involvement of NGB in other biological processes and/or its contribution to other cellular compartments is undetermined.

Here, we report the quantitative changes in the proteome of ERα-positive human breast cancer cells (namely, the MCF-7 cell line) with the *NGB* gene knocked out using CRISPR/Cas9 technology. The MCF-7 cells have been successfully used as a subject in different essential proteomic investigations [10,11,12]. Accordingly, the effect of different treatments (E2, H_2_O_2_, and E2 + H_2_O_2_) on the proteome of the MCF-7 cell line is reported here. Taking advantage of comparative proteomic investigations combined with multiple statistical and bioinformatic analyses, several cellular pathways that are mainly related to cytoskeleton dynamics, response to stress, apoptosis, and mitochondria-driven processes were found to be unbalanced in NGB-deficient cells. These processes may take place in breast cancer cells in the absence of NGB, especially when NGB expression is not inducible via the administration of NGB-inducible factors.

## 2. Results

### 2.1. Comparative Proteomic Analysis of NGB-KO and Neg MCF-7 Cells

**Figure 1:**
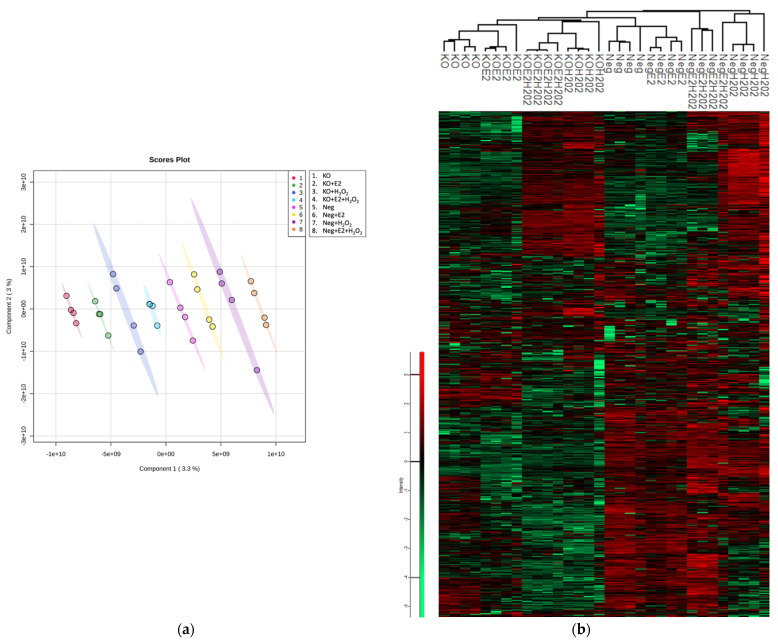
Statistical separation of the analyzed groups and distribution of the quantitative data. (**a**) PLS-DA was performed, showing good separation between the eight experimental groups. Each experimental group is labeled with a numbered class color and each number corresponds to a specific group. (**b**) The heatmap was generated to visualize the distribution of protein abundances throughout the replicates using adjusted data based on Z-score normalization. The green and red color ranges refer to the lower and higher abundances, respectively.

Neuroglobin knockout (NGB-KO) was obtained in MCF-7 cells using CRISPR/Cas9 technology, as previously described [13]. In addition, a negative control (Neg) clone was obtained using the same procedure [13]. To study the altered biological pathways and processes related to the NGB deficiency in MCF-7 cells, the variation in protein profile was quantitatively assessed using shotgun LFQ (label-free quantification) proteomics in NGB-KO vs. Neg cells. Furthermore, the proteomic investigation was carried out in NGB-KO and Neg MCF-7 cells upon treatment with (i) E2 (10 nM, 24 h), (ii) H_2_O_2_ (400 μM, 24 h), and (iii) E2 + H_2_O_2_ (E2 10 nM, 4 h pretreatment; 400 μM H_2_O_2_, 24 h), as previously reported [7]. Thus, after liquid chromatography–tandem mass spectrometry (LC–MS/MS) analysis and comparison of the matching groups, a total of four individual proteomic datasets were obtained. Protein abundances were calculated in each condition using an LFQ algorithm in the MaxQuant software. To evaluate the quality of the LC–MS/MS runs, normal distributions of the data were assessed within the Perseus framework (Supplementary Figure S1a) and adjusted via mean-centered scaling (Supplementary Figure S1b). The statistical separation of the normalized datasets was analyzed using partial least squares—discriminant analysis (PLS-DA), leading to the complete separation of the eight analyzed groups (Figure 1a). Finally, a global overview of protein abundances distribution along each biological replicate was visualized using a heatmap (Figure 1b).

**Figure 2:**
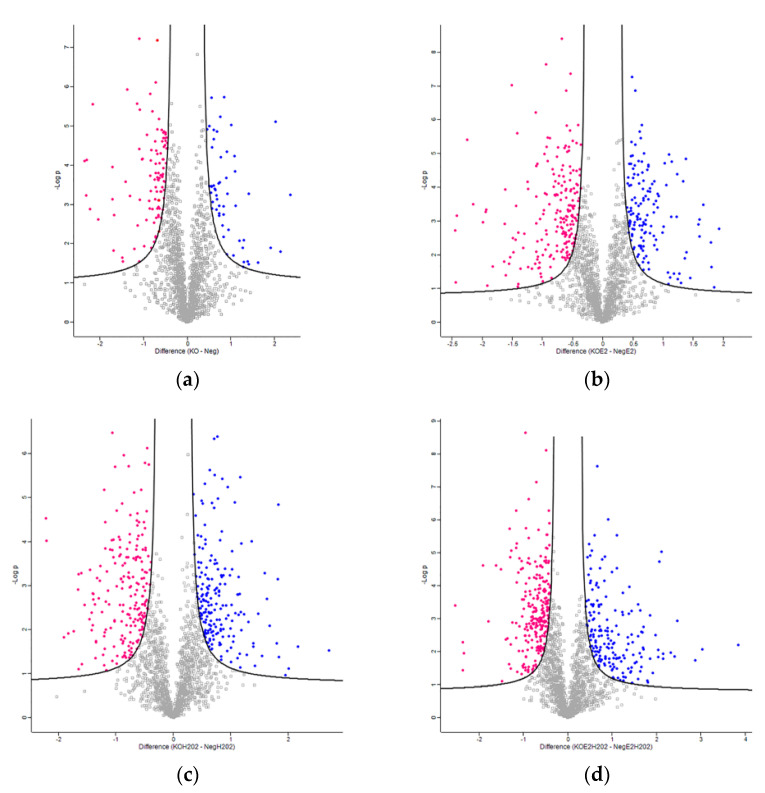
Global proteome distribution of the four experimental conditions. Volcano plots report the protein fold-change (indicated as Difference) against the statistical significance (–log *p*) in (**a**) NGB-KO vs. Neg, (**b**) NGB-KO + E2 vs. Neg + E2, (**c**) NGB-KO + H_2_O_2_ vs. Neg + H_2_O_2_, and (**d**) NGB-KO + E2 + H_2_O_2_ vs. Neg + E2 + H_2_O_2_. The blue and pink dots represent the up- and downregulated proteins, respectively.

The global proteomic dataset obtained consisted of 3072 proteins that were identified using a number of peptides >1 in at least one sample. Protein quantification was performed on 1872 proteins that showed valid values in the 70% over all the runs. The protein fold change for each treatment condition was calculated as a log2 difference of the means of protein intensity in the NGB-KO and Neg replicates. To select the statistically significant proteins, the two-sample *t*-test was applied. The statistically significant proteins with a fold change ≥0.4 (upregulated) and ≤−0.4 (downregulated) are reported in Table 1, and graphically as volcano plots (Figure 2). Supplementary Tables S1–S4 contain the lists of the differentially regulated proteins for each treatment condition.

### 2.2. Bioinformatic Analysis for the Functional Enrichment of Proteomics Data

The differential proteome of the nontreated NGB-KO cells was analyzed using the DisGeNET platform to discover potential gene–disease associations (Table 2). Within the regulated dataset, 76 proteins were recognized as belonging to the “breast carcinoma” class, showing the lowest *p*-value (1.25 × 10^−10^). In agreement with the significant association of the differential proteins with cancer, other cancer-related classes were enriched from the analysis as well. Thus, the DisGeNET tool confirmed that most of the differential proteins were recognized as being related to breast carcinoma.

**Figure 3:**
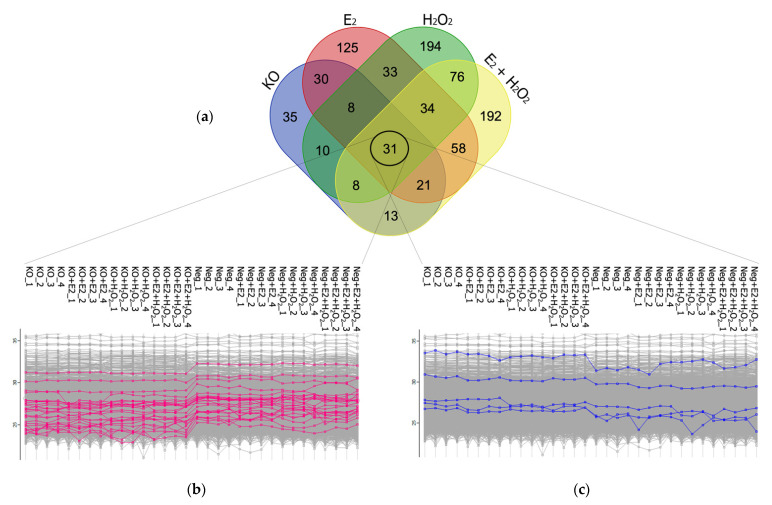
Eulero–Venn analysis of the differentially regulated proteins in the four analyzed conditions focusing on common proteins. (**a**) The Eulero–Venn analysis returned 31 regulated proteins as being in common for all the conditions. All the proteins showed the same trend of abundance throughout the replicates, except for one that was discarded. In detail, (**b**) 25 out of 30 proteins showed low abundance while being downregulated in all the NGB-KO conditions, and (**c**) 5 out of 30 proteins showed high abundance while being upregulated in all the NGB-KO conditions.

**Figure 4:**
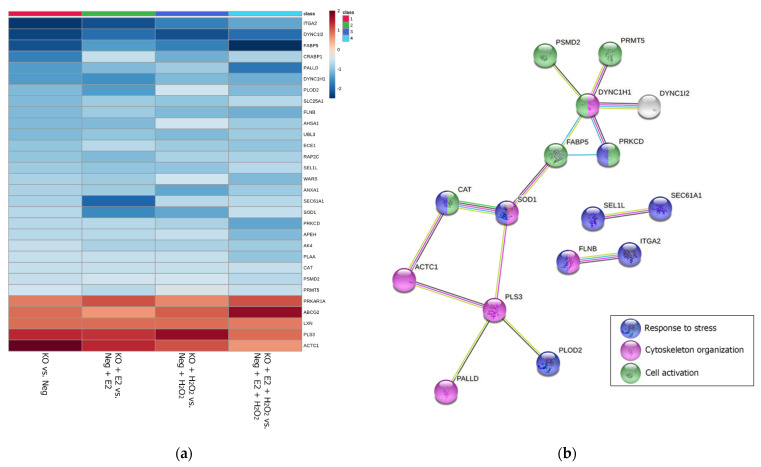

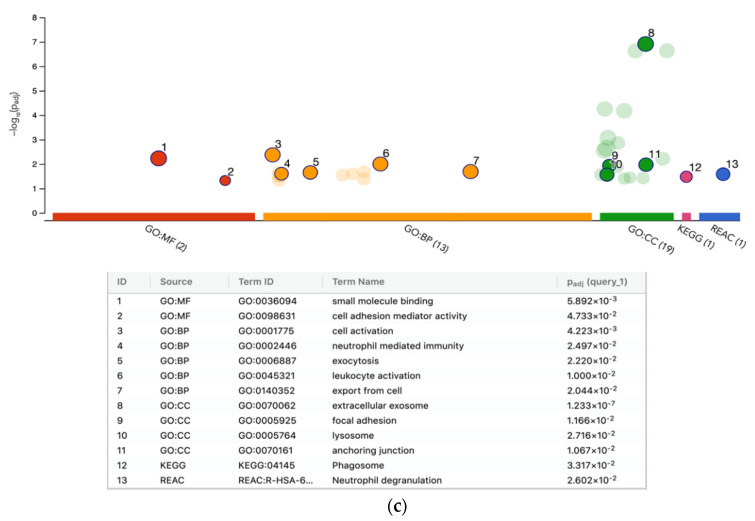
Heatmap, STRING network, and functional profiling of the common protein dataset. (**a**) The heatmap shows the distribution of the protein fold changes in the four experimental conditions. The blue and red color ranges refer to the lowest and the highest fold changes, respectively. Each experimental group is labeled with a numbered class color and each number corresponds to a specific group. (**b**) The STRING network was built using the connecting nodes from the common protein dataset; the labeled nodes refer to the biological processes represented by the highest counts in the network. (**c**) The analysis using g:Profiler enriched the significant non-redundant GO terms (MF—molecular function, BP—biological process, CC—cellular component), KEGG, and REACTOME pathways. The box shows the details of the enriched categories.

Furthermore, since a specific treatment (E2, H_2_O_2_, or E2 + H_2_O_2_) may cause a specific biological response in NGB-KO cells, the regulated proteomes from the four conditions were intersected using an Eulero–Venn analysis to select common and exclusive features (Figure 3). Common elements should be addressed to the knockout gene background, exclusively. Accordingly, 31 proteins were found to be regulated in all four conditions. The protein abundances were checked throughout the replicates and 30/31 proteins showed the same trend of regulation in all the conditions (Table 3 and Figure 4a). The protein with discordant quantitative behavior (PRKDC) was discarded from the bioinformatic enrichment analysis of the common protein dataset but included in Table 3 (marked in red). As shown in Figure 3, the pink and blue dots represent the LFQ intensities of the down- and upregulated proteins, respectively, in all NGB-KO vs. Neg (without and with treatments) comparisons. Thus, bioinformatic functional profiling was performed using the 30-protein dataset in the STRING and g:Profiler tools. The analyses enriched the processes that were common to all four conditions that were possibly imputed by the knockout gene background, such as the response to stress, cytoskeleton organization, and cell activation for STRING, and neutrophil and leucocyte activation, exocytosis and export from the cell, or alterations related to cell junction, focal adhesion, and exosome and lysosome compartments for g:Profiler (Figure 4b,c).

**Figure 5:**
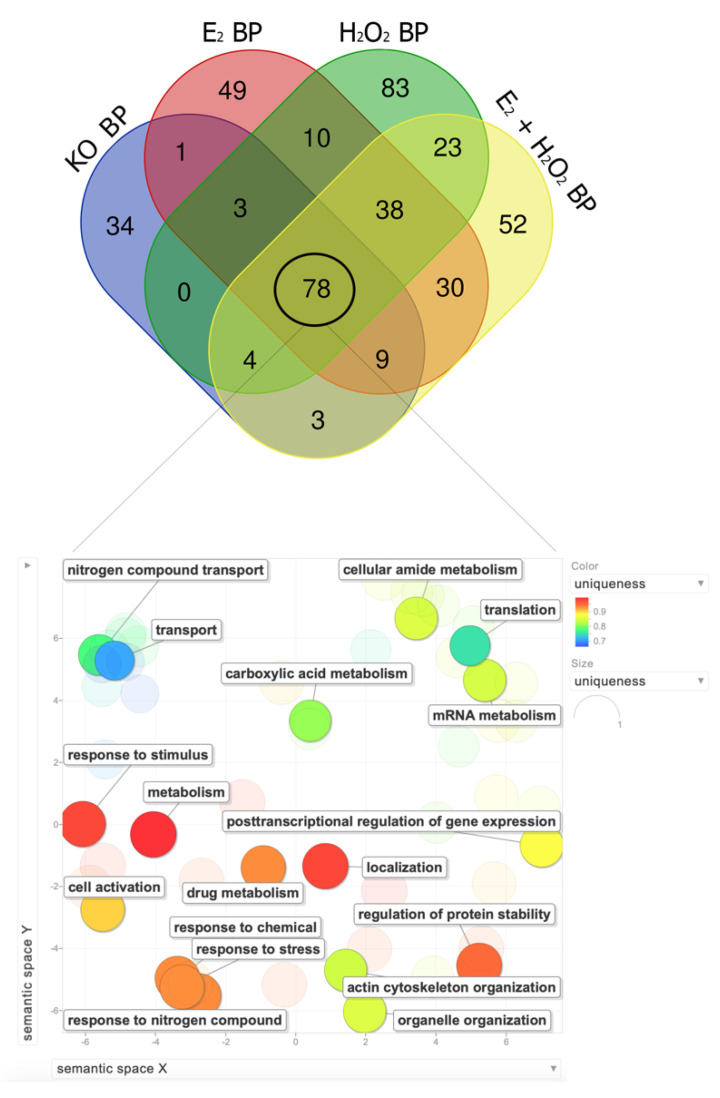
Eulero–Venn analysis of the BPs identified in the four analyzed conditions with a focus on the common BPs. The Eulero–Venn analysis returned 78 BP that were common to all the conditions. At the bottom of the figure, the significant non-redundant BPs are shown. The color grade of the BPs refers to the semantic uniqueness of the terms, ranging from blue (uniqueness = 0.7) to red (uniqueness = 1).

Then, each regulated proteome underwent independent functional enrichment using the STRING and the REViGO tools. Eulero–Venn analysis was performed to focus on the biological processes (BPs) identified in all the conditions with a *p*-value < 0.01 (Figure 5). The four conditions shared 78 common BPs that, as already mentioned, should be imputed to the knockout background. From these 78 BPs, the significant non-redundant BPs were selected (Figure 5). The enrichment of the common BPs found from the global proteomes was performed to confirm the analysis of the BPs of the small common dataset. Accordingly, the analysis highlighted the dysregulation of proteins involved in response to stress, cell activation, actin cytoskeleton organization, and organelle organization in all the analyzed conditions. Intriguingly, with the NGB being involved in scavenging reactive nitrogen species (RNS) and reactive oxygen species (ROS) [14], the NGB knockout also induced the alteration of BPs related to the response to the metabolism of nitrogen compounds (Figure 5).

**Figure 6:**
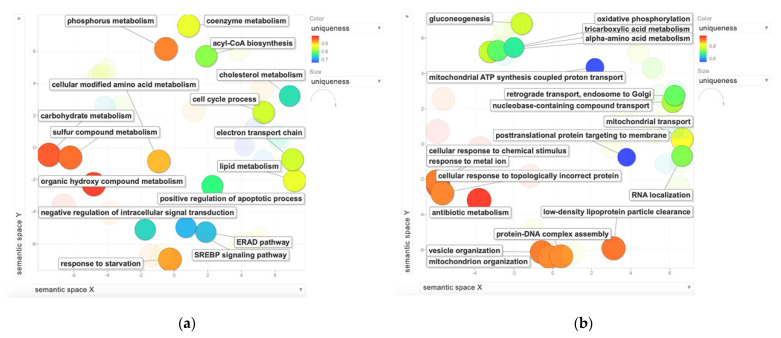

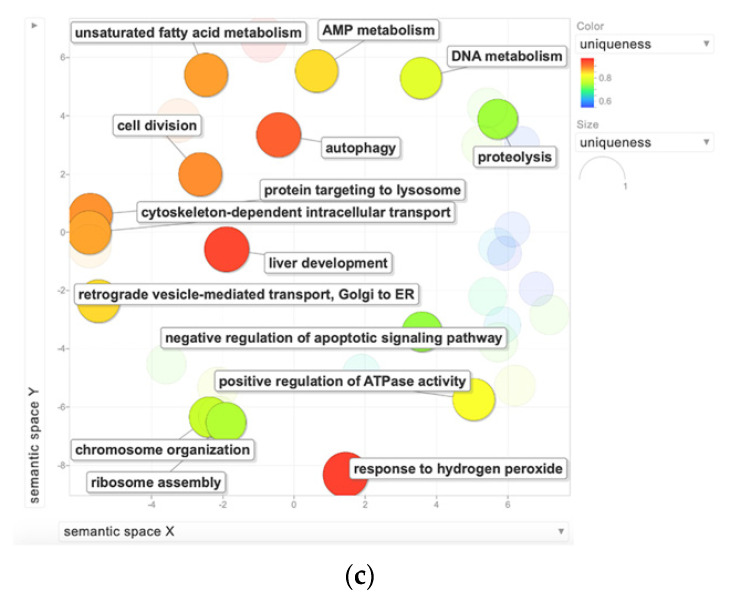
BPs that were specifically identified in the three treated NGB-KO conditions. Significant and non-redundant BPs were selected using REViGO software in (**a**) NGB-KO + E2 vs. Neg + E2, (**b**) NGB-KO + H_2_O_2_ vs. Neg + H_2_O_2_, and (**c**) NGB-KO + E2 + H_2_O_2_ vs. Neg + E2 + H_2_O_2_. The color grade of the BPs refers to the semantic uniqueness of the terms, ranging from blue (uniqueness = 0.6 or 0.7) to red (uniqueness = 1).

Finally, to discover specific terms that were correlated with the specific treatment, the above-described common BPs were subtracted from the independent bioinformatic analyses of the NGB-KO cells treated with E2, H_2_O_2_, and E2 + H_2_O_2_. Moreover, the terms detected using the common proteins dataset (Figure 4c) were subtracted as well. The resulting non-redundant BPs were specifically enriched for each considered category and are reported in Figure 6.

## 3. Discussion

NGB is a relatively recently discovered protein [1] that displays neuroprotective effects that are exerted in neurons after its overexpression as an outcome of several stimuli, such as hypoxic events, oxidative stress, and oxygen/glucose deprivation [2,9]. Mounting interest in NGB function has recently been growing in non-neuronal compartments and tissues, such as breast cancer cells, highlighting the antioxidant and prosurvival functions of E2-induced NGB expression [3]. By being expressed endogenously at very low levels, many NGB-overexpressing and NGB-deficient models have been generated with the aim to induce insults on the system studied and, subsequently, investigate the NGB response. In vivo NGB-deficient models showed ambiguous results that do not support the neuroprotective hypothesis for endogenous NGB, as NGB overexpression models do [15]. NGB deficiency in mice exacerbated the response to hypoxia mediated by Hif1A and c-FOS, without affecting neuronal survival [16]. In rats, NGB silencing impaired the respiratory chain complexes I and III activity, and the retina and the visual function [17]. By contrast, in vitro NGB-deficient cellular models assign a role for endogenous NGB in the defense against oxygen and glucose deprivation, oxidative stress, and apoptosis, supporting retinal function and neuronal development [15]. Furthermore, ambiguous results have been reported in cancer cells, where NGB knockdown promoted cell growth or functioned as a tumor suppressor [18,19].

To the best of our knowledge, the current study represents the first proteomic survey that was performed in a cellular model of human breast cancer deficient in NGB [15]. In particular, the goal was to highlight unbalanced pathways when human NGB is not expressed and/or not inducible (for example by E2 or H_2_O_2_).

The present analysis highlighted the regulation of a small dataset of proteins, whose quantitative variation occurred under all the experimental conditions (also with the administration of E2 and/or H_2_O_2_), reflecting the *NGB* gene knockout background. Indeed, the subsequent bioinformatic analysis enriched several processes that were imputed to the NGB knockout. The alteration of BPs under all the conditions reinforced their importance and their consideration for widening the knowledge about NGB function in the cell. From our results, STRING analysis enriched cell activation, response to stress, and cytoskeleton organization were found as common BPs throughout the treatment conditions.

Particularly, cell activation refers to “a change in the morphology or behavior of a cell resulting from exposure to an activating factor such as a cellular or soluble ligand” (http://amigo.geneontology.org/amigo/term/GO:0001775, accessed on 7 March 2021) [20]. In an astrocytic model under rotenone insult, NGB upregulation preserves the mitochondrial morphology [21]. Instead, the “mitochondrial organization” of the BPs was altered in MCF-7 cells upon H_2_O_2_ treatment when NGB was knocked out. The main effects on mitochondrial biological processes, such as “ATP synthesis coupled to proton transport”, “tricarboxylic acid metabolism”, and “oxidative phosphorylation”, were more evident in H_2_O_2_-treated MCF-7 cells. In fact, it has been suggested that NGB may play a role in mitochondrial ATP production [4,22]. In the SH-SY5Y cell line, the decrease in the oxidative stress caused by H_2_O_2_ and the increase in the levels of ATP were driven by the upregulation of NGB levels [22,23]. In addition, H_2_O_2_ treatment highlighted several cellular responses, such as “cellular response to chemical stimulus”, ”response to metal ion”, ”post-translational protein targeting to membrane”, and “cellular response to topologically incorrect protein”. This is coherent with the effects produced by ROS action that may interfere with proteins regulating their levels, by either modulating gene expression or by modifying their structure and stability [24].

Endogenous NGB was proved to be a ROS-inducible protein in MCF-7 cells and a key factor for E2-induced breast cancer progression [7]. E2 increases cell survival by preventing mitochondrial-dependent apoptosis through the activation of ERα in E2-dependent cancer cells. Similarly, our bioinformatic analysis of MCF-7 cells treated with E2 showed “positive regulation of apoptotic process” and the alteration of proteins involved in the “cell cycle process”, suggesting a lacking inhibition of the apoptotic cascade due to the absence of NGB. By contrast, in the cells treated with E2 + H_2_O_2_, the enrichment of the ”negative regulation of apoptotic signaling pathway” occurred, suggesting that apoptosis may be inhibited despite NGB not being expressed in these cells. On the other hand, the presence of BPs such as “autophagy”, “protein targeting to lysosome”, “proteolysis”, and “cell division” may suggest a more complex response to the combined treatment for the survival/death fate of these breast cancer cells. Autophagy may be upregulated as a consequence of stress or nutrient deprivation [25,26]. Of note, autophagy can protract the survival of cancer cells defecting in apoptosis [27].

Despite divisive findings and speculations on the role of E2 on autophagy [28,29,30,31,32], the E2 + H_2_O_2_ combination may specifically modulate the autophagic flux. Nevertheless, NGB overexpression does not affect the modulation of autophagy regulators [33], although the effects of NGB knockout on autophagy have not been tested yet.

Since the main role of NGB is ascribed to the protection of the cell from stress stimuli, such as hypoxia and oxidative stress, the “response to stress” BPs were coherent and of great interest. This class includes the following downregulated proteins: superoxide dismutase 1 (SOD1), catalase (CAT), PLOD2, ITGA2, FLNB, PRKCD, SEL1L, and SEC61A1. Redox homeostasis in cancer cells is aberrant and its regulation may be under the control of the cell antioxidant defense based on SOD1, CAT, and the glutathione system. Furthermore, the persistent action of an oxidative stimulus in cancer cells induces cell adaptation to the redox stress, potentiating the expression of these protective systems [3,24]. Similarly, it was demonstrated that NGB displays a direct role in the adaptation of cancer cells to the increased oxidative stress by several mechanisms, including ROS scavenging and the potentiation of the antioxidant response [3,13]. Thus, as a consequence of the NGB knockout, the quantitative abundance of enzymes like SOD1 and CAT decreased compared to the control cells. Accordingly, when the (over)expression of NGB did not occur because of gene deletion, the “response to stress”, “response to nitrogen compounds”, and “nitrogen compound transport” BPs were commonly enriched in the treated NGB-KO cells as well. This reinforced the role of NGB as a key modulator of the oxidative response since the levels of the above-mentioned enzymes remained downregulated, even in the proteome of E2- and H_2_O_2_-treated cells. Of note, most of the proteins included in the class of “response to stress”, “cell activation”, and related to neutrophil and leucocyte activation are recognized by REACTOME [34] as part of the immune system pathway (PLAA, FLNB, PRKCD, FABP5, PRKDC, CAT, DYNC1H1, DYNC1I2, PSMD2, RAP2C, SEC61A1, ANXA1, SOD1, and APEH) that is downregulated in the absence of NGB. Therefore, NGB may act in cancer cells as a functional hub that allows for the crosstalk between oxidative stress and inflammation pathways, which has recently been called “oxinflammation” [35].

In all the analyzed proteomes, cytoskeletal proteins were found to be subjected to quantitative variation (mostly being downregulated) as a common feature of NGB-deficient breast cancer cells. Cytoskeleton dynamics is a prerequisite for changing cancer cell morphology and for acquiring an invasive and migratory phenotype, and for the epithelial to mesenchymal transition (EMT) process [36]. In this context, even though the treatment of MCF-7 cells with exogenous NGB does not evidence promigratory effects [9], the present data open the intriguing possibility that the positive modulation of intracellular NGB levels can take part in the promotion of breast cancer cell motility through the activation of ERα [37,38]. Furthermore, it has been demonstrated that the overexpression or the knockdown of cytoskeleton-related proteins augments or decreases, respectively, the release of exosomes [39], which resulted as a CC enriched in MCF-7 NGB-deficient cells throughout the treatment conditions. The modulation of cytoskeleton proteins and extracellular exosomes in the NGB knockout background, together with data indicating that NGB is differently released in response to E2 and H_2_O_2_ [9], might suggest that this globin is linked to the cancer cell ability to modify the extracellular microenvironment. Indeed, on the one hand, NGB is a dynamic component of the cancer cell secretome that can affect the “response to stress” on neighbor cancer and non-transformed breast cells from the outside, eliciting their adaptation to microenvironmental stresses [9]. On the other hand, the intracellular NGB can take part in the regulation of breast-cancer-dependent shaping of the extracellular milieu in response to external stimuli.

Furthermore, NGB upregulation improves the actin condensation induced by H_2_O_2_, suggesting preservation of cell membrane integrity and mitochondrial transportation [23]. Because the actin cytoskeleton and actin-binding proteins are required for mitochondrial morphology, motility, and immobilization of the organelle at the cell cortex [40], mounting evidence suggests an indirect function of NGB in mitochondrial transportation [22]. In addition, NGB appears to be involved in the protection from hypoxia by interfering with cytoskeletal polarization and lipid-raft-dependent death signaling within the Rho GTPase pathway [41]. All the above-mentioned processes seem to be lacking in MCF-7 cells in the absence of NGB.

## 4. Materials and Methods

### 4.1. Cell Culture

The MCF-7 biallelic *NGB* gene knockout (NGB-KO) and its negative control (Neg), which were genetically modified using the CRISPR/Cas9 technology, were obtained from GenScript Corporation (Piscataway, NJ, USA) and validated as previously reported [13] (Supplementary Figure S2). The MCF-7 Neg and MCF-7 NGB-KO cell lines were used from four to eight passages and were grown in air containing 5% CO_2_ and DMEM without phenol red medium. The medium contained 10% (*v/v*) fetal bovine serum, gentamicin (0.1 mg/mL), l-glutamine (2 mM), and Pen-strep solution (penicillin 100 U/mL and streptomycin 100 mg/mL). The cells were simultaneously treated with the vehicle used to dissolve all drugs (ethanol/PBS 1:10, *v/v*), and/or E2 (10 nM, 24 h), or H_2_O_2_ (400 μM, 24 h) in the presence or absence of the E2 pretreatment (10 nM, 4 h).

### 4.2. Proteome Extraction and S-Trap Tryptic Digestion

The cells were collected and processed as described in [42,43,44]. Briefly, the cell samples were lysed in RIPA buffer (Sigma-Aldrich, St. Louis, MO, USA). Then, lysates were treated with 1% Benzonase (E8263-5KU, Sigma-Aldrich, St. Louis, MO, USA) plus 2 mM MgCl_2_ and incubated at 37 °C for 30 min to degrade all the nucleic acids (DNA and RNA), and centrifuged at 18,000 rpm for 30 min at 4 °C. The supernatants were collected and the protein concentration was determined using the Bradford assay. Protein digestion was performed on 50 µg of each sample after adding a final concentration of 5% SDS. The reduction was carried out with 10 mM TCEP (Sigma-Aldrich, St. Louis, MO, USA) and carbamidomethylation of cysteines with 40 mM iodoacetamide (Sigma-Aldrich, St. Louis, MO, USA). Protein digestion was performed on an S-Trap™ micro spin column (Protifi, Huntington, WV, USA) using Sequencing Grade Modified Trypsin (Promega, Madison, WI, USA) at 47 °C for 1 h. The digested peptides were eluted from the S-Trap columns, vacuum dried, and kept at −80 °C until analysis.

### 4.3. LC–MS/MS Analysis

Dried samples were resuspended in 100 µL of 10% ACN, 0.1% TFA in HPLC-grade water, and injected (1 µL) into an RSLC Ultimate 3000 chromatograph coupled with a Q Exactive PLUS mass spectrometer (Thermo Scientific, Waltham, MA, USA). LC–MS/MS analysis was performed as reported in [43]. Briefly, peptide mixtures were loaded onto a µ-precolumn (Acclaim PepMap 100 C18, cartridge, 300 µm i.d. × 5 mm, 5 µm), and then separated on a 50 cm reversed-phase liquid chromatographic column (0.075 mm ID, Acclaim PepMap 100, C18, 2 µm) (both Thermo Scientific, Waltham, MA, USA). Solvents for the chromatographic separation were 0.1% formic acid in water (A) and 80% ACN and 0.08% formic acid (B). The following gradient was set to elute peptides from the column: from 5 to 40% B (180 min), from 40 to 80% (1 min); 80% (5 min); fixed at 5% for 20 min for column re-equilibration. Blanks were run between samples to prevent sample carryovers. Eluting peptides underwent fragmentation using higher-energy collisional dissociation (HCD). MS/MS analysis was performed via data-dependent acquisition (DDA) using a top 10 method. Q Exactive PLUS had a resolution set at 70,000 for the MS scans and 17,500 for the DDA MS/MS scans in order to increase the speed. An MS scan range from 400 to 2000 *m/z* was set. The MS automatic gain control (AGC) target was set to 3 × 10^6^ counts with a maximum injection time of 60 ms, while the MS/MS AGC target was set to 1 × 10^5^ with a maximum injection time of 60 ms; the dynamic exclusion was 30 s. Four independent biological replicates per condition were analyzed.

### 4.4. Label-Free Quantification Analysis

The MaxQuant software (version 1.6.3.4) was employed for processing the .raw MS files [45]. The Andromeda search engine worked using the UniProt *Homo sapiens* reference proteome (Proteome ID: UP000005640; Taxonomy: 9606—Homo sapiens). Default features within MaxQuant were selected. Trypsin was set as the proteolytic enzyme, allowing for up to two missed cleavage sites, and a length of seven amino acids was set as the minimum peptide length. The fixed modification was the carbamidomethylation of cysteine, and the variable modifications were the oxidation of methionine and *N*-term protein acetylation. The MaxQuant LFQ algorithm was used for protein quantification. The false discovery rate (FDR) was set to 1% at both the protein and peptide levels, and a match between runs was selected. Then, the MaxQuant-generated protein groups file was loaded onto Perseus software (version 1.6.0.7), which was used for statistics and data elaboration. The dataset was reduced by removing contaminants, reverse hits, and proteins only identified by site. After a log2-transformation of the LFQ data, only proteins identified by a number of unique peptides >1 in 70% over all of the runs were retained. The imputation of missing values was performed by selecting a downshift of 1.8 and a width of 0.3 standard deviations in a Gaussian distribution of random numbers. A normal distribution of protein intensity from each LC–MS/MS run was assessed. Then, the normal distributions of the data were adjusted using mean-centered scaling and the separation of the experimental groups was validated using PLS-DA by employing MetaboAnalyst 5.0 software [46,47]. After the data preprocessing, the protein fold changes were calculated as the log2 protein difference of the intensity means of the NGB-KO and Neg replicates groups. Differentially regulated proteins were selected using a two-sample *t*-test (FDR = 1%, S0 = 0.2). Proteins with a fold change ≥0.4 (upregulated) and ≤−0.4 (downregulated) were selected. Volcano plots and profile plots were generated within Perseus [48,49]. The heatmap for the visualization of the protein abundances of the common protein dataset was generated with MetaboAnalyst software. To get an overview of the protein intensities from the global proteomic dataset, the heatmap was built within Perseus using the adjusted data based on Z-score normalization.

### 4.5. Bioinformatics Analysis

To reveal alterations of the protein expression profiles of MCF-7 NGB-KO cells, multiple bioinformatic analyses were performed. The DisGeNET platform within EnrichR software [50,51] was investigated to retrieve gene–disease associations. The functional profiling of a set of proteins common to the four analyzed conditions was obtained using g:Profiler software [52,53]. The enrichment of Gene Ontology (GO) Biological Process (BP) terms for each regulated proteome was performed using STRING [54,55,56]. The long lists of GO-BPs obtained from STRING were reduced and summarized using the REViGO software, with the final selection of the significant non-redundant BP terms [57,58].

## 5. Conclusions

Here, we report for the first time a proteomic survey in NGB-deficient human breast cancer cells. The present findings shed light on biological processes that were affected by NGB expression and on the regulation of intracellular globin levels. Indeed, BPs that were commonly altered in the absence of NGB throughout the different conditions belonged to the apoptotic regulation, mitochondrial organization, and the large class of “response to stress,” reinforcing the idea of critical functions of inducible NGB in such events [5,6,8,13]. Furthermore, the herein reported results open a new scenario over NGB’s role in breast cancer cells, providing evidence of its possible involvement in further critical cellular processes, including cytoskeleton dynamics and extracellular microenvironment shaping. Even though the role of NGB in such events needs to be further validated, these data lead to the consideration of a wide and complex function of NGB as a hub protein that regulates biological processes, with a particular focus on those processes that are activated in response to external hormonal and/or stress stimuli. Remarkably, if a similar imbalance pertaining to these intracellular processes occurs in the absence of NGB, as well as in other cellular contexts (i.e., neurons), it is worth evaluating. Indeed, such evidence would clarify whether the reported biological processes that were affected by NGB occur as a common feature or whether they are cell/tissue-specific, opening new possibilities in the definition of NGB-related targetable pathways.

## Figures and Tables

**Table 1:** Qualitative results of the differentially regulated proteomes after the comparative analysis.

Experimental Condition	Total Differential Proteins	Downregulated Proteins	Upregulated Proteins
NGB-KO vs. Neg	156	102	54
NGB-KO + E2 vs. Neg + E2	340	202	138
NGB-KO + H_2_O_2_ vs. Neg + H_2_O_2_	395	204	191
NGB-KO + E2 + H_2_O_2_ vs. Neg + E2 + H_2_O_2_	433	252	181

**Table 2:** DisGeNET gene–disease associations in NGB-KO cells.

Term	Overlap	*p*-Value
Breast carcinoma	76/4963	1.25 × 10^−10^
Malignant neoplasm of breast	75/5054	6.05 × 10^−10^
Carcinogenesis	66/4065	9.53 × 10^−10^
Neoplasm metastasis	63/3920	1.69 × 10^−9^
Malignant neoplasm of prostate	53/3239	3.76 × 10^−8^
Spinal muscular atrophy	12/196	4.59 × 10^−8^
Malignant neoplasm of the lung	44/2449	6.16 × 10^−8^
Primary malignant neoplasm of the lung	41/2268	1.72 × 10^−7^
Carcinoma of the lung	43/2476	2.40 × 10^−7^
Lymphoma	29/1307	2.75 × 10^−7^

**Table 3:** Common protein dataset in all NGB-KO comparisons with fold changes.

		Protein Fold Change			
UniProt ID	Gene Name	NGB-KO vs. Neg	NGB-KO + E2 vs. Neg + E2	NGB-KO + H_2_O_2_ vs. Neg + H_2_O_2_	NGB-KO + E2 + H_2_O_2_ vs. Neg + E2 + H_2_O_2_
P17301	ITGA2	−2.4	−2.2	−1.7	−1.3
Q13409	DYNC1I2	−2.3	−1.9	−2.2	−1.9
Q01469	FABP5	−2.2	−1.4	−1.7	−2.5
P29762	CRABP1	−1.7	−0.6	−1.2	−0.8
Q8WX93	PALLD	−1.4	−1.1	−0.9	−1.8
Q14204	DYNC1H1	−1.4	−1.5	−1.1	−1.2
O00469	PLOD2	−1.1	−1.4	−0.5	−1.1
P53007	SLC25A1	−1.1	−0.9	−1.0	−0.7
O75369	FLNB	−1.1	−0.9	−1.1	−1.2
O95433	AHSA1	−1.1	−1.1	−0.5	−0.9
O95164	UBL3	−1.1	−1.0	−1.1	−0.9
P42892	ECE1	−1.0	−0.7	−0.9	−1.0
Q9Y3L5	RAP2C	−1.0	−1.1	−0.8	−0.8
Q9UBV2	SEL1L	−0.9	−1.0	−1.0	−0.7
P23381	WARS	−0.8	−0.9	−0.5	−1.1
P04083	ANXA1	−0.8	−0.9	−1.3	−0.9
P61619	SEC61A1	−0.8	−2.0	−0.7	−0.7
P00441	SOD1	−0.7	−1.6	−1.3	−0.6
Q05655	PRKCD	−0.7	−0.7	−0.8	−1.3
P13798	APEH	−0.7	−0.7	−0.6	−1.1
P27144	AK4	−0.6	−0.8	−0.8	−0.9
Q9Y263	PLAA	−0.6	−0.6	−0.6	−1.0
P04040	CAT	−0.6	−0.6	−0.6	−0.6
Q13200	PSMD2	−0.6	−0.5	−0.5	−0.7
P78527	PRKDC	− 0.6	− 0.4	+0.6	+0.8
O14744	PRMT5	−0.5	−0.7	−0.4	−0.6
P10644	PRKAR1A	+0.8	+1.1	+0.7	+1.1
Q9UNQ0	ABCG2	+0.9	+0.6	+1.0	+1.7
Q9BS40	LXN	+0.9	+0.9	+0.9	+0.8
P13797	PLS3	+1.4	+1.3	+1.7	+0.9
P68032	ACTC1	+2.0	+1.4	+1.1	+0.6

The red-marked protein refers to the single protein of the common dataset with discordant quantitative behavior throughout the experimental conditions.

## Data Availability

The mass spectrometry proteomics data have been deposited to the ProteomeXchange Consortium via the PRIDE partner repository with the dataset identifier PXD023734.

## Supplementary Materials

The following are available online. Supplementary Figure S1: Statistical distribution of the proteomics data, Supplementary Figure S2: NGB levels in CRISPR/Cas9 knockout MCF-7 cells, Supplementary Table S1: List of the differentially regulated proteins in the NGB-KO vs. Neg proteome, Supplementary Table S2: List of the differentially regulated proteins in the NGB-KO + E2 vs. Neg + E2 proteome, Supplementary Table S3: List of the differentially regulated proteins in the NGB-KO + H_2_O_2_ vs. Neg + H_2_O_2_ proteome, Supplementary Table S4: List of the differentially regulated proteins in the NGB-KO + E2 + H_2_O_2_ vs. Neg + E2 + H_2_O_2_ proteome.

## Sample Availability

Samples of the compounds used in this study are available upon request from the authors.
