# Supplementary material for: Proteomic and Bioinformatic Investigation of Altered Pathways in Neuroglobin-Deficient Breast Cancer Cells
Source: Molecules. 2021 Apr 20;26(8):2397. doi: 10.3390/molecules26082397 (PMC8074618; doi:10.3390/molecules26082397)
Supplement: Supplementary file 1 [file molecules-26-02397-s001.zip › molecules-1159227-supplementary/Supplementary Figures.pdf]

Supplementary Materials

Proteomic and Bioinformatic Investigation of Altered Pathways in Neuroglobin-Deficient Breast Cancer Cells

Michele Costanzo [1,2], Marco Fiocchetti [3], Paolo Ascenzi [3], Maria Marino [3], Marianna Caterino [1,2,*] and Margherita Ruoppolo [1,2,*]

1 Department of Molecular Medicine and Medical Biotechnology, School of Medicine, University of Naples Federico II, 80131 Naples, Italy; michele.costanzo@unina.it
2 CEINGE—Biotecnologie Avanzate s.c.ar.l., 80145 Naples, Italy
3 Department of Science, University Roma Tre, 00146 Rome, Italy; marco.fiocchetti@uniroma3.it (M.F.); paolo.ascenzi@uniroma3.it (P.A.); maria.marino@uniroma3.it (M.M.)
* Correspondence: marianna.caterino@unina.it (M.C.); margherita.ruoppolo@unina.it (M.R.)

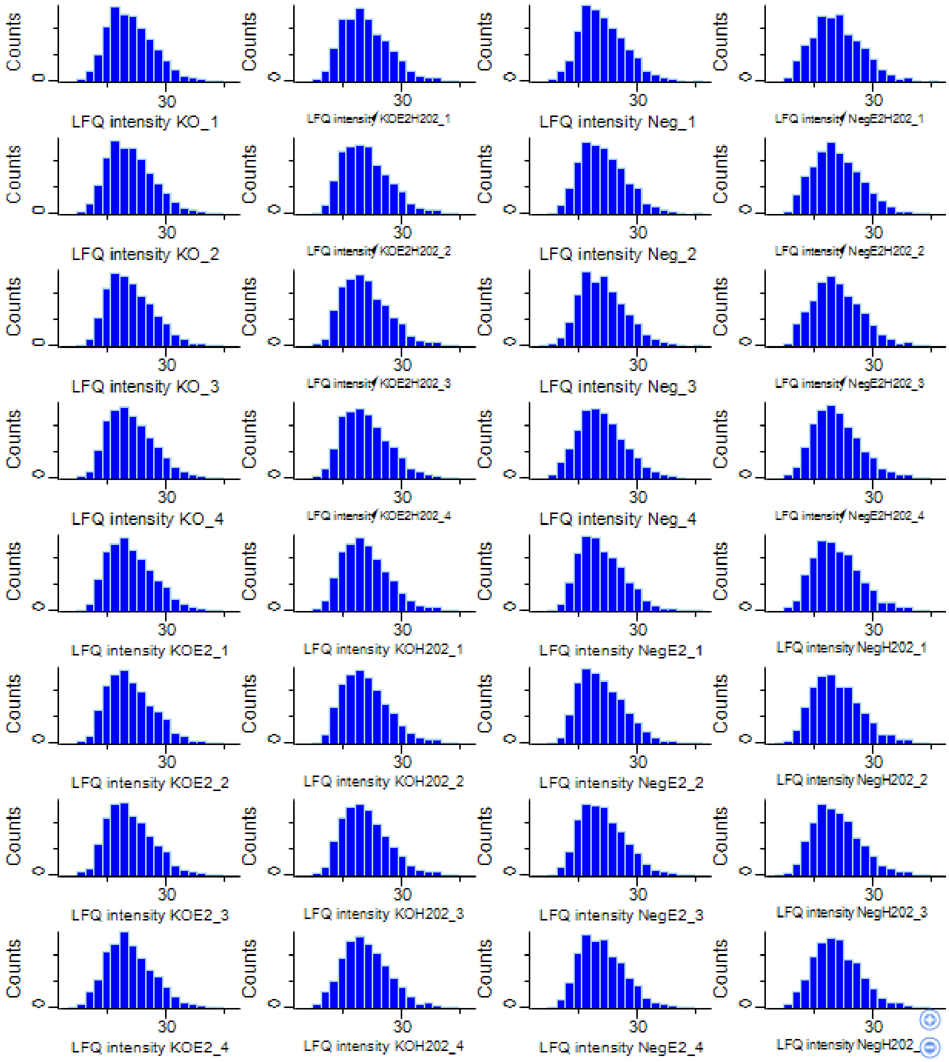

(a)

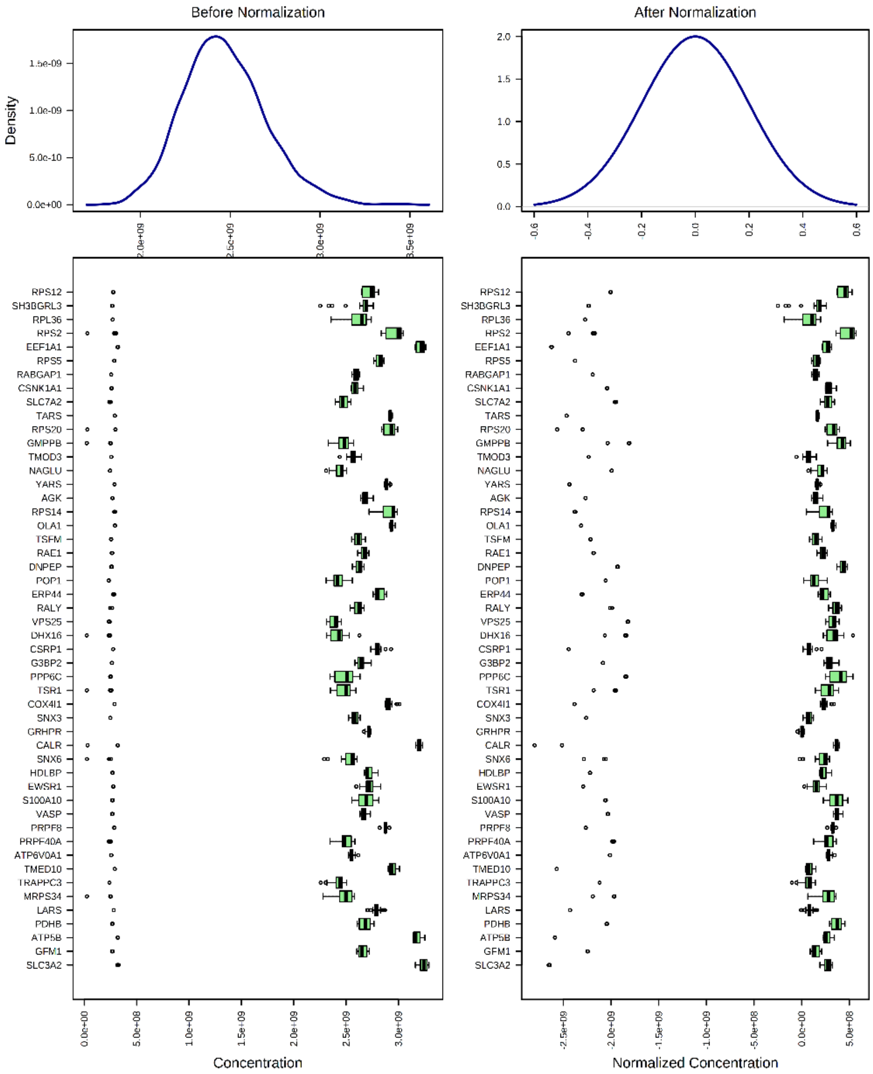

(b)

Figure S1. Statistic distribution of proteomics data. **(a)** Normal distribution of LFQ values of each LC-MS/MS run was evaluated with Perseus software plotting LFQ intensities as histogram bars; the plot for each biological replicate/condition/treatment is shown. **(b)** Normal distribution of data was adjusted by mean-centered scaling within MetaboAnalyst 4.0 software.

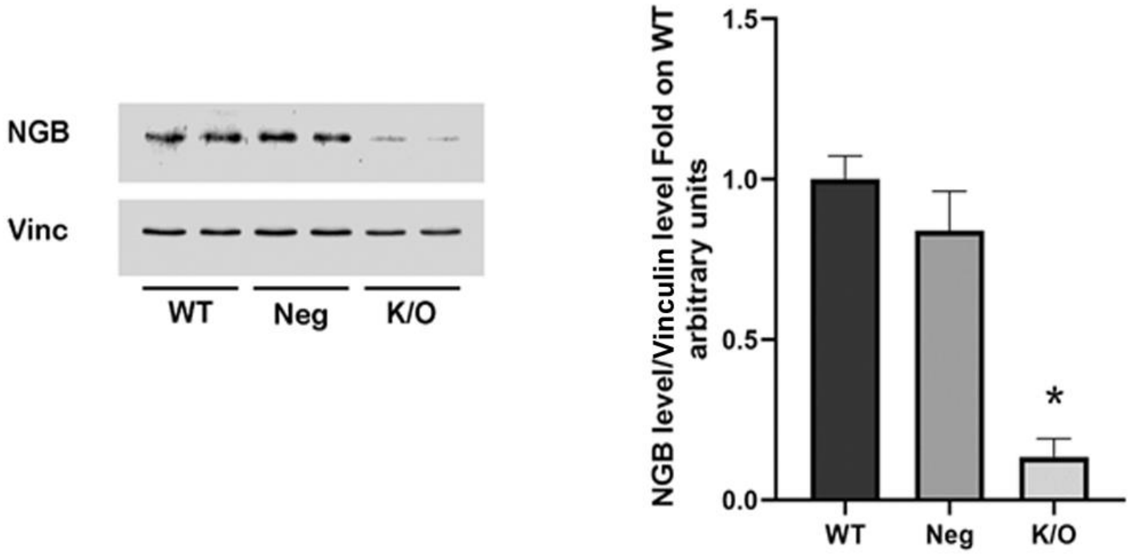

Scheme S2. NGB levels in CRISPR/Cas9 knockout MCF-7 cells. Representative Western Blot (left panel) and densitometric analysis (right panel) of NGB level in Wild Type (WT), Negative (Neg) and NGB-KO (K/O) MCF-7 cells. The levels of NGB proteins were normalized by comparison with vinculin levels. Western blot analysis was performed as previously described (*Solar Fernandez, V.; Cipolletti, M.; Ascenzi, P.; Marino, M.; Fiocchetti, M. Neuroglobin As Key Mediator in the 17β-Estradiol-Induced Antioxidant Cell Response to Oxidative Stress. Antioxid. Redox Signal.* ***2020****, 32, 217–227, doi:10.1089/ars.2019.7870*). Cells were harvested and lysed with a buffer mix (50 mM HEPES at pH 7.5, 10% glycerol, 150 mM NaCl, 1% Triton X-100, 1 mM EDTA, 1 mM EGTA) containing 0.70% (*w*/*v*) SDS. Fifteen μg of solubilized proteins were resolved by 13.5% SDS-PAGE transferred into a nitrocellulose membrane using the Trans-Blot Turbo Transfer System (Bio-Rad Laboratories, Hercules, CA, USA) for 7 min at 25V. The membrane was then blocked with 5% (*w*/*v*) BSA in 138mM NaCl, 25mM Tris, pH 8.0, and 0.1 (*w*/*v*) Tween-20 at 25 °C for 1 h, and then incubated overnight at 4 °C with anti-NGB antibody (final dilution 1:1000, MERK Millipore, Darmstadt, DE), and anti-Vinculin antibody (1:40000, MERCK Millipore, Darmstadt, DE) used to normalize the protein content. Antibody reactivity was observed with ECL chemiluminescence Western blotting detection reagent by using ChemiDoc XRS+ Imaging System (Bio-Rad Laboratories, Hercules, CA, USA). The densitometric analyses was performed by ImageJ software for Microsoft Windows (NIH, Bethesda, MD, USA). The statistical analysis was performed by Student's t-test with the PRISM 6.01 software system (GraphPad Software, Inc, San Diego, CA, USA) for Windows. Data are means ± SD of at least three different experiments. * = $p < 0.05$.
